# A New Health Networking Infrastructure on Cancer Is Taking Shape in Europe: A Not-to-Miss Opportunity for the EU Regulatory System

**DOI:** 10.3390/jmahp13020018

**Published:** 2025-04-28

**Authors:** Paolo Giovanni Casali, Stefano Capri

**Affiliations:** 1 Medical Oncology Unit 2, Fondazione IRCCS Istituto Nazionale Tumori, 20133 Milan, Italy; paolo.casali@istitutotumori.mi.it; 2 Department of Oncology and Haemato-Oncology, Università degli Studi di Milano, 20100 Milan, Italy; 3 School of Economics and Management, Cattaneo-LIUC University, 21053 Castellanza, Italy

At the inaugural EAA convention in Copenhagen (Europe’s Evolving HTA Regulation and Its Relevance for ‘Beating Cancer’, in May 2022), it was suggested that, in the future, the pillars necessary to fully address the problem of cancer should be communicated (i.e., the comprehensive cancer centres, JANE, and the EU’s HTA endeavour). This Editorial represents a development in those themes, and to some extent, it represents two sides of the same coin, i.e., the clinical and the HTA sides. In 2021, the European Union (EU) launched the EU Beating Cancer Plan [1]. This is a Europe-wide initiative by which the EU sets ambitious targets for the following years in order to improve cancer survival. Among the many efforts foreseen by this initiative, there was the creation of a European network of comprehensive cancer centres and the creation of new “Networks of Expertise” (NoEs). Two EU Joint Actions started therefrom, in order to pave the way for the creation of such networks; additionally, two follow-up Joint Actions are currently in progress with the aim of deploying such networks. The networks of European comprehensive cancer centres will be called EUNetCCC. There will be seven Networks of Expertise on the following items: survivorship; palliative care; poor-prognosis cancers; economic technologies (i.e., technologies that are based on the ability to process large amounts of biological data, as the whole set of genes in genomics, etc.); high-tech medical resources; personalized primary and secondary prevention; and adolescents and young adults with cancer. These networks will be created within the aforementioned Joint Actions, while four other European networks have been in place since 2017 in the area of rare cancers: the European Reference Networks (ERNs) EURACAN, EuroBloodNet, PaedCan, GENTURIS, respectively, on rare adult solid cancers, haematological neoplasms, paediatric cancers, and hereditary conditions predisposing to cancer [2]. It is well known that rare cancers are marked by difficulties in delivering appropriate care as well as in conducting clinical studies, thus resulting in health discrimination [3,4,5]. Health networks have a special role in addressing such challenges.

Therefore, in the future, the EU will be supported by 12 networks focused on cancer. At the moment, some of them are split up into domains that are networks themselves, as long as they pursue different tasks and are rooted in diverse communities. Aside from the 10 domains of EURACAN (each corresponding to one rare adult solid cancer), the NoE on high-tech medical resources will be split up into domains covering innovative radiation therapy, nuclear medicine, radiomics, innovative surgery, ablative techniques, cell therapies, and ex vivo testing, while the NoE on poor-prognosis cancer will be split up into at least two domains on pancreatic cancer and lung cancer. It follows that the EU is deploying a formidable array of health networks in the cancer arena.

The strength of this approach is that healthcare networking can well become a peculiar European asset. Clinical collaboration may be more difficult in other areas of the globe; therefore, the tool of formalized health networks can display great potential. Several opportunities can be brought about by these networks, including diminishing disparities among EU member states (MSs); sharing health data, possibly within the new EU *Health Data Space* (HDS); fostering clinical and translational research; and contributing to medical, patient, and public education and awareness. It is worth noting that networks can contribute to the improvement of prescription choices in anticancer drugs, whose economic impact is enormous. Global spending on cancer medicine increased to USD 223 billion in 2023, USD 25 billion more than in 2022, and it is projected to reach USD 409 billion by 2028 [6].

The only threat to these networks is that they may miss such a huge goal amid their numerous potential weaknesses. It is no coincidence that the first Joint Action on Networks of Expertise on Cancer (JANE) ended its preparatory effort by publishing a “green paper” raising several questions about the future of all EU health networks [7]. Some questions had to do with how to best implement health networking, exploring and improving its cost/effectiveness (in the end, it is noteworthy that the medical literature is lacking in research on health networking tools). On top of that, while ERNs were launched by the EU Commission under the legislative tool of the EU Cross-Border Healthcare Directive, both EUNectCCC and the NoEs will be launched through the tool of the two Joint Actions. In any case, not all ERNs will be legal entities, thus preventing them from being able to do several things, among which are sponsoring clinical trials, raising funds, etc. This may also impact how these networks will be able to collect and process health data in a legal framework that is greatly affected by how European and national data protection authorities interpret the EU General Data Protection Regulation (GDPR), thereby impairing the ambitions of the HDS. In addition, this is in an operational framework in which the interoperability of electronic health records is still a dream, albeit in the age of artificial intelligence (AI) [8]. Another open question has to do with the relationship of these networks with the industry. The main open questions, however, have to do with how the Europe-wide and national levels can complement each other and give rise to an interconnected system of European and national networks acting together. Ideally, these European networks should become “networks of networks”, i.e., European networks of national/regional networks (at least in regard to the largest MSs). This is challenging within the well-known context of a union whose founding treaties do not incorporate health. Thus, ambitions are great, but weaknesses are manifold and need to be tackled, as long as these networks grow. Finally, network activities could positively contribute to direct prescriptive choices in anticancer drugs that also take into account the economic aspects of treatments, considering the enormous economic impact of such drugs. Indeed, global spending on cancer medicine increased to USD 223 billion in 2023, which is USD 25 billion more than in 2022, and this is projected to reach USD 409 billion by 2028 [6].

All this said, while trying to effectively answer the open questions raised by the JANE green paper, one may wonder which opportunities may be implied by the development of those networks under the regulatory perspective, with regard to both anticancer drugs and medical devices. We list some of them here.

- At the very least, these networks will gather an incredible amount of available expertise from throughout Europe. Sometimes, the impression of regulatory bodies is that they fail to collect all the expertise that would be needed to deal with technologies that are evolving at an incredible pace. While networks are able to convey methodological skills as well as, say, pharmacological expertise, they may find it more difficult to not only deal with rapidly evolving medical technologies but also to recognize the actual clinical needs posed by very rare cancers. While clinical research methodology has failed to keep up with such an impressive pace of technological progress (today, clinical studies may often be outdated even before they are closed), regulatory challenges are huge. Relying on such new communities, each focusing on highly specialized technological or clinical areas, may become an incredible asset for regulatory bodies to exploit. Bringing disease-based expertise to the appropriate regulatory authorities would mean closing the gap between regulators, industry, and clinical and patient experts; currently, regulators provide “scientific advice” to companies, and the latter interact with clinical researchers, but a link between regulators and clinical and patient experts is often missing. In fact, all these networks have been conceived so as to accommodate the patient’s perspective by effectively involving patient advocacy groups. Some of them are disease-based, so they can bring about highly specific expertise on the patient’s side. Thus, such networks can well convey to regulatory bodies the kind of patient expertise that they need. Conflicts of interest may be managed more effectively within independent networks deployed by the EU.
- All of these networks will be endorsed by the EU and its MSs. This means, in a sense, that they are trusted by national governments and that they may be in a position to interact effectively with national health systems. Some regulatory tools, for example, may be underexploited due to a lack of interaction between the EU and the national level. One example of a regulatory tool may be “adaptive licensing”, by which a drug might be given the temporary approval to be continuously updated according to the latest knowledge that such a temporary approval might allow the generation of [9]. Clearly, this would require high levels of cooperation between the EU and individual nations. For example, ERNs on rare cancers could well be a privileged framework within which to try such innovative solutions. One may also think of the enormous issue of the off-label use of drugs in oncology, which is handled with highly diverse, and sometimes irrational, solutions throughout the EU, without generating any new knowledge. The controlled off-label use of drugs, at least for rare cancers, by means of ERNs, could become an opportunity to improve patient access to some anticancer agents while concurrently generating new evidence through governed efforts.
- Amid the difficulties of classic randomized clinical trials in keeping pace with evolving technologies, there is a huge need for external controls in uncontrolled studies, as well as for frameworks within which to generate reliable health data that are liable to be processed with AI. Networks naturally lend themselves to being exploited to this end. They may easily develop shared standard operating procedures among a lot of institutions, making quality control easier and diminishing the costs of research while simultaneously complying with all regulatory requirements set in advance. Attempts at some degree of relaxation of data protection rules within such public health endeavours may become possible.
- Even if these networks are not legal entities, thus being unable to sponsor clinical trials, their health institutions can do it. Thus, they could become privileged frameworks to at least stimulate academic, independent clinical trials. There is a huge need for post-approval independent trials, and all the more so at a time of the fast conditional approval of new drugs based on uncontrolled studies and/or limited patient samples. In fact, many new drugs in oncology are approved on the basis of surrogate endpoints, while data on overall survival are still incomplete or immature. For example, of 223 cancer drug indications approved by the FDA in 2001–2018, 95 had an overall survival as an endpoint, and of these, 41% had immature survival data [10]. After a minimum of 4–3 years of follow-up during the period after marketing authorization, only in 32% of indications did additional data on overall survival show any statistically significant benefit. Effective collaborations could be established between regulatory bodies and academic systems through all these networks. Information, and possibly studies, suggested by networks can help review the value of EMA decisions regarding oncology drugs.
- In a recent study [11], 131 oncology drugs approved by the EMA with 166 indications were evaluated for their added benefits within the required time frame in at least one HTA organization, resulting in a total of 458 assessments of added benefits. Among the 458 assessments of added benefits, 41% were negative or unquantifiable. Drugs with a higher added benefit valuation generally have higher revenues. Negative or unquantifiable ratings of added benefits were more common for the conditional approvals and approvals under exceptional circumstances than for marketing authorizations of standard marketing. While revenues appear to align with added benefits, most oncology drugs recover R&D costs within a few years, despite providing few added benefits. This is especially true for drugs approved through conditional marketing authorizations, which seem inherently lacking in comprehensive evidence.

The authors believe that the potential of such virtuous circles could hardly be exaggerated. Clearly, the weaknesses of these networks, as put forth by the JANE green paper, may be a limiting factor. On the other hand, early successes might stimulate the EU to upscale the status of these networks, at a time when it is very clear, much beyond healthcare, that many EU rules may need to be revised in order to be competitive in a challenging world.

Thus, what is needed now is the study of networks—how to best exploit their potential, how to direct their action, and how to maximize their cost/effectiveness should become areas of interest for a highly multidisciplinary audience of researchers. This could make the most of a peculiar European asset. Then, the scientific community, along with patient communities, should interact with regulators in order to envisage the best forms of cooperation. The political level can then be involved in an attempt to foster discussions about how to fix some of the most obvious weaknesses of the current EU health networks. Challenges to this approach are not easy to take on, but it is definitely worth it, and this is also true from a regulatory perspective.
